# Comorbidity, Pain, Utilization, and Psychosocial Outcomes in Older versus Younger Sickle Cell Adults: The PiSCES Project

**DOI:** 10.1155/2017/4070547

**Published:** 2017-03-28

**Authors:** Donna K. McClish, Wally R. Smith, James L. Levenson, Imoigele P. Aisiku, John D. Roberts, Susan D. Roseff, Viktor E. Bovbjerg

**Affiliations:** ^1^Department of Biostatistics, Virginia Commonwealth University, Richmond, VA 23298, USA; ^2^Department of Internal Medicine, Virginia Commonwealth University, Richmond, VA 23298, USA; ^3^Department of Psychiatry, Virginia Commonwealth University, Richmond, VA 23298, USA; ^4^Department of Emergency Medicine, Harvard University, Boston, MA 02115, USA; ^5^Department of Internal Medicine, Yale University, New Haven, CT 06520, USA; ^6^Department of Pathology, Virginia Commonwealth University, Richmond, VA 23298, USA; ^7^College of Public Health and Human Sciences, Corvallis, OR 97331, USA

## Abstract

*Background*. Patients with SCD now usually live well into adulthood. Whereas transitions into adulthood are now often studied, little is published about aging beyond the transition period. We therefore studied age-associated SCD differences in utilization, pain, and psychosocial variables.* Methods*. Subjects were 232 adults in the Pain in Sickle Cell Epidemiology Study (PiSCES). Data included demographics, comorbidity, and psychosocial measures. SCD-related pain and health care utilization were recorded in diaries. We compared 3 age groups: 16–25 (transition), 26–36 (younger adults), and 37–64 (older adults) years.* Results*. Compared to the 2 adult groups, the transition group reported fewer physical challenges via comorbidities, somatic complaints, and pain frequency, though pain intensity did not differ on crisis or noncrisis pain days. The transition group utilized opioids less often, made fewer ambulatory visits, and had better quality of life, but these differences disappeared after adjusting for pain and comorbidities. However, the transition group reported more use of behavioral coping strategies.* Conclusion*. We found fewer biological challenges, visits, and better quality of life, in transition-aged versus older adults with SCD, but more behavioral coping. Further study is required to determine whether age-appropriate health care, behavioral, or other interventions could improve age-specific life challenges of patients with SCD.

## 1. Introduction

Sickle cell disease (SCD) is an autosomal recessive genetic disorder of hemoglobin structure whose classic symptomatic manifestations are acute episodes of ischemic pain, termed vasoocclusive crises (VOC), from deformed red blood cells. VOC may begin within the first year of life and generally continue throughout life. VOC worsen with age [1–3] even among children [4, 5]. VOC may often require costly emergency care, hospitalization, and/or opioids for relief [6, 7].

Fortunately, newborn screening [8], prophylactic penicillin [9], screening for stroke, and prevention of stroke with red cell transfusions [10] and hydroxyurea [11–14] have put a huge dent in the previously high SCD morbidity and mortality rate in childhood [15], from deadly infections and strokes and in adulthood from vasoocclusive complications and organ failure. While in 1970 the estimated median survival for people with SCD was just 20 years [16], today many with SCD are living well into adulthood. Perhaps the first generation to have many survivors past age 40, the majority of living SCD patients are now adults, with a median age at death over 40 [17, 18]. Still, adults with SCD face unique challenges related to mounting comorbidities of organ failure as well as the general challenges of aging [19–23]. There are well-documented age-related changes in hospital utilization throughout the lifecycle [6].

Not surprisingly, the adolescent transition period to adulthood has become an intense focus of health services and implementation research in SCD, because it is fraught with difficulty. Many have described the challenges of transition to adulthood from pediatric care [24–28]. Like all adolescents and young adults, SCD patients must learn to manage the uncertainties of transitioning out of their homes of origin, obtaining training and education, and seeking employment if they are healthy enough. But in addition, SCD transition-age patients must learn to manage their chronic disease, negotiate care on their own with providers, and cope with an often stark, more disjointed adult health care system, compared to the pediatric system. Further, hospitals face ballooning utilization of emergency and hospital services by SCD patients during the adolescent transition period [29–31]. Behavioral and other interventions are being tested to improve the transition from pediatric to adult SCD care, such as peer mentoring, teaching self-care, or improving patient activation [32–37].

However, in contrast, few have studied age-specific life challenges, including biological challenges and psychosocial challenges, beyond the transition period, that is, among the oldest SCD patients. Most previous studies of utilization and their predictors among adult SCD patients were done with adults with an average age of 30 years [38]. It is conceivable that if age-specific challenges were found in these older SCD adults, specific interventions could be crafted to improve the quantity and quality of their lives, similar to what are being developed for transition-aged patients.

We are therefore interested in several age-associated differences among adults with SCD. We theorized three age groups by stage of life: transition-aged, younger adults, and older adults. Building off of ours [39] and others' earlier theories we hypothesized that older SCD patients, due to aging and organ failure, would report more comorbidities, have worse laboratory measures, and report worse functional status and more health care utilization than the younger adults or transition-aged groups. On the other hand, we hypothesized that those in the transition-aged group would, due to more disruption in social support and living conditions, utilize services more than slightly older, presumably better-adjusted, but still “young” adults, who might be enjoying their “stable, family” years. Finally, due to more comorbid illness in the older adult groups, we hypothesized that each of these groups might report more pain, more psychosocial dysfunction, and poorer coping than the younger adult group.

## 2. Methods

PiSCES (the Pain in Sickle Cell Epidemiology Study) was a longitudinal study of pain in SCD but also a methodologic study of the relationship among measures of pain, crises, and utilization in sickle cell disease. The methods of PiSCES have been described in detail elsewhere [40, 41]. Briefly, we enrolled patients from July 2002 to August 2004. We collected baseline information including demographic characteristics and medical history, physical and mental health related quality of life (HRQOL), depression, alcoholism, somatic symptom burden, sickle cell-related stress, coping styles, and social support. We collected laboratory data via blood and urine samples. Patients then kept daily pain diaries for up to 6 months, including names and amounts of opioid-containing medications taken.

We recruited patients 16 years of age or older from across Virginia, mostly from the Richmond (central Virginia) and Tidewater (coastal) areas. Both the study and our recruitment methods were approved by the Institutional Review Board of Virginia Commonwealth University, Richmond, Virginia, and we obtained informed consent.

Patients received routine care for their sickle cell disease from either community-based physicians or sickle cell specialist physicians associated with academic medical centers (two physicians at Virginia Commonwealth University Health System serving the Richmond area and one physician associated with Eastern Virginia Medical School, Norfolk, Virginia, serving the Tidewater region). Emergent care for the cohort was provided in Emergency Departments regardless of the patients' usual source of ambulatory care. No day hospitals for sickle cell disease are located in the region.

### 2.1. Measures

Coping was assessed using the Coping Strategy Questionnaire-SCD (CSQ-SCD), originally developed to measure cognitive and behavioral coping styles in chronic ow back pain [42]. This scale was later revised for patients with SCD with the addition of items related to strategies particularly relevant to SCD [38]. The adapted Coping Strategy Questionnaire-SCD includes 78 items (each rated on Likert scale from 0 to 6). While there are 13 subscales of 6 items each, we followed the methods and results of Anie et al. [43] (also confirmed by our own factor analysis) and used the 3 scales: active coping (ignoring pain sensations, reinterpreting pain sensations, calming self-statements, diverting attention, and increasing behavioral activities), affective or emotional coping (anger, fear, catastrophizing, praying and hoping, and isolation), and passive or behavioral adherence coping (taking fluids, resting, and heat/cold/massage). Scores are means of the subscales.

The Smith-Bovbjerg Sickle Cell Stress scale (STRESS) is a 10-item scale that assesses stress using our internally developed measure (Cronbach's alpha 0.84). More specifically, items are measured on a Likert scale from 0 (strongly disagree) to 4 (strongly agree) and assess issues as worry about access to pain medication or whether pain medication will control pain, being hospitalized, limited work activities, sexual performance, insurance, and so on. Higher scores indicate more perceived stress.

Health related quality of life was assessed at baseline using the Medical Outcome Study 36-item short form (MOS SF-36), one of the earliest well-known and accepted generic measures of HRQOL [44]. There are eight SF-36 subscales measured on a scale from 0 to 100 (worst to best) as well as two summary scales of physical and mental health. The SF-36 has good reliability and validity in subjects with chronic pain [45, 46]. We have reported overall SF-36 results of PiSCES elsewhere [47].

Social Support was measured using the Multidimensional Scale of Perceived Social Support [48, 49]. Patients rated 12 items on a scale from 1 to 7 regarding the amount of support received from family, friends, and significant others. Higher mean values indicate better perceived social support.

Depression, anxiety, alcohol abuse, and somatic symptoms were each measured using the Patient Health Questionnaire (PHQ). The PHQ is a widely utilized screening instrument based on subjects' self-reported symptoms that was designed to facilitate the recognition and diagnosis of the most common mental disorders in primary care subjects [50]. For the purposes of this study, we combined the two depression diagnoses generated by the PHQ (Major Depressive Syndrome, Other Depressive Syndrome) and combined the two anxiety diagnoses (Panic Syndrome, Other Anxiety Syndrome) into a single category of depression/anxiety [51]. The designation of alcohol abuse was also derived from the PHQ [52].

Somatic symptom burden was assessed with the portion of the PHQ referred to as the PHQ-15 [53], which includes 15 physical symptoms (like headaches, dizziness, heart pounding, sleep problems, nausea, bowel function issues, etc.) that account for more than 90% of symptoms seen in primary care (exclusive of upper respiratory symptoms such as cough). Patients with higher somatic symptom scores have been found to use more health care (both inpatient and outpatient) and this relationship remains strong after controlling for presence of comorbid psychiatric conditions. In order to avoid measuring overlapping physical symptoms which could be accounted for by SCD instead of somatization, we excluded four common pain sites of SCD (limb, back, stomach, and chest) and used an 11-item version referred to as PHQscd [54].

Daily pain assessment utilized daily diaries completed by patients for up to 6 months. Patients were encouraged at the initial baseline visit and with reminder calls by study staff to return diaries daily by mail using provided stamped envelopes. They received payment for each returned diary, with a higher payment in the latter 2 months of the study to encourage study completion. We modeled the diary after that of the Multicenter Study of Hydroxyurea (MSH) [55]. Patients reported about the prior 24 hours. They rated their worst sickle cell pain intensity (0–9) and marked a body diagram to indicate where they hurt [56]. In addition, they reported whether they were in a “crisis” and whether they had made a visit to the emergency department (ED) and hospital or either a scheduled or unscheduled clinic visit because of sickle cell pain. Patients also indicated what medication they had taken. Crisis days were self-defined by each patient using a check box on each daily diary.

### 2.2. Statistical Analysis

For purposes of analysis, we defined home days as days without ED or hospital utilization marked on the diary and pain days as days when pain intensity was rated 1 or higher on the 0–9 scale. We defined opioid use as the report of use of any of the known opioids then on the market, inclusive of Tramadol.

Mean scores of pain intensity as well as rates of pain occurrence, health care utilization, and home opioid use were calculated for each patient using daily diary information. Since the number of diaries completed by each subject varied, the numbers of pain days, opioid use days, crisis days, and utilization days were converted to percentages by dividing by the total number of diary days completed by each subject. The exception was the percentage of home pain days on opiates, which used home pain days as the denominator. The denominator for mean pain was pain days. For mean pain on crisis days we used number of diary days reporting crisis in the denominator, while for mean pain on noncrisis days the denominator was number of pain days without crisis.

A measure called ED reliance (referred to here as ED reliance–health care) was calculated as the proportion of ambulatory visits (ED + outpatient visits) that are ED visits [57]. This measure is said to be able to distinguish between ED visits due to lack of adequate access to primary care versus increased need. A value greater than .33 is considered high. An additional ED reliance related measure (referred to as ED reliance–crisis) was created as the proportion of total number of crises that were treated at the ED/hospital as opposed to at home.

Guided by our cited hypotheses, as well as by Sanders et al. [58], who compared 70 adults 18–36 versus a group aged 37 and above, we divided patients into 3 age groups: transition (ages 16–25), younger adults (ages 26–36), and older (ages 37 and above) adult groups. Categorical measures were compared using a Chi-square test; Bonferroni correction was used when pairwise comparisons were made. Continuous measures were compared across these 3 age categories using analysis of variance. In certain cases we controlled for percent pain days and number of SCD comorbidities. Tukey's method was used to compare age categories pairwise while controlling for multiple comparisons in ANOVA and ANCOVA.

Analyses were conducted by using SAS, version 9.4 for Windows (SAS Institute, Cary, North Carolina).

### 2.3. Role of the Funding Source

The National Heart, Lung, and Blood Institute provided an unrestricted grant for this project. The funding source did not have any influence on the planning, conduct, analysis, or publication of this study or its results.

## 3. Results

Table 1 shows that a total of 57 subjects were in the transition group, (ages 16–25), 71 subjects were in the younger adult group (ages 26–36), and 104 subjects were in our older adult group (ages 37–64). Compared to the two adult groups, the transition group was less likely to have gone to college or be married, likely a direct effect of their age/stage in life. The two adult groups had similar education levels. Income and genotype were similar across age groups. A similar proportion of subjects among each age group attended specialty medical care centers for SCD.

Subjects in both the transition group and the younger adult group recalled significantly fewer comorbidities and less often reported ischemic ulcers, hypertension, or rheumatic diseases over their lifetime compared to the older group. Subjects in the transition group also less often recalled avascular necrosis and gout than did those in the adult groups (specific comorbidities are reported in Supplemental Table  1 in Supplementary Material available online at https://doi.org/10.1155/2017/4070547). Subjects in the transition group were less likely to be depressed/anxious than either of the adult groups, but this difference did not persist when controlling for frequency of pain and comorbidities.

Table 2 shows that the subjects in either the younger or older adults had nearly twice the percentage of pain days, had twice the average pain intensity among all days, and hurt at nearly one-and-a-half times as many body sites as those in transition group. However, subjects did not differ with regard to the percent of days spent in crisis (though there was a numeric trend), or with regard to mean pain intensity on crisis days or noncrisis pain days.

Regarding health care and opioid utilization, Table 3 shows that, before controlling for pain days and comorbidities, the transition group had fewer outpatient visits and used opioids on fewer days than did the older adult group. When controlling for pain days and comorbidities, differences in outpatient visits among age groups were no longer significant (2.7% versus 2.7% versus 2.9%, *p* = 0.9179). There were no significant differences for ED visits, hospital days, or total health care utilization. Notably, ED reliance for health care was twice as high for either the transition group or younger adult group (which had similar values) as compared to the older adult group. With values greater than 33 percent, the two younger age groups had EDR which would be considered excessive [29]. The ED reliance for crisis was not significantly different when comparing the unadjusted averages, but, after adjusting for comorbidities and percent pain days, the transition group used the ED/hospital for a crisis rather than self-care at home significantly more often than the two adult groups (23.3 versus 13.8 versus 9.1, *p* = 0.0237). There was also a trend for the younger adult group to use the ED/hospital for a crisis more than the older adult group.

Table 4 shows comparisons among age groups for psychosocial variables and HRQOL. The transition group reported significantly fewer somatic symptoms than the younger or older adult groups, although these differences were no longer significant when controlling for pain days and comorbidities (6.2 versus 7.2 versus 7.5, *p* = 0.1123). The transition group also reported using significantly less passive-adherence coping than the younger adults. This difference persisted when controlling for pain days and comorbidities.

The transition group reported significantly better physical health (SF-36 physical summary score), which persisted when controlling for pain days and comorbidities. All individual SF-36 subscales were significantly better for the transition group than for the two older groups, except for mental functioning, where there were no significant differences across age groups. When controlled for pain frequency and comorbidities, vitality, physical functioning, and physical role function subscales were still significantly better for the transition group (individual subscales in Supplemental Table 2). There were no differences between age groups in reports of social support or SCD stress.

## 4. Discussion

Although our results are not the first to compare age groups of patients with SCD with regard to disease or psychosocial outcomes, our results are some of the first to compare simultaneously age differences in measures of physical outcomes, utilization, pain outcomes, and psychosocial outcomes of SCD adults. Our goal was to further test hypotheses about important theoretical subthemes and potential predictors of utilization of care and pain in SCD that we raised in 1997 [40].

For this study, we hypothesized age-related changes in these predictors and outcomes. Regarding outcomes, we hypothesized that pain, utilization (health care and opioid), and psychosocial outcomes of older SCD adults were sometimes even worse than those for transition-aged adults. Regarding predictors, we hypothesized worse organ failure (worse laboratory measures) and comorbidities for older adults. We were aware that transition-aged adults were already well-documented as having worse utilization of health care, life disruption [6, 30], and HRQOL [59] than pediatric patients. We therefore hypothesized that disruption in social support and living conditions would be worse for transition patients than for older adults and that this would lead to transition patients utilizing services more than older, presumably better-adjusted adults, who might be enjoying their “stable, family” years. Our analyses confirmed some but not all of our hypotheses. We found some age-related differences in all of the domains we measured: biomedical, utilization, pain, functional, and psychosocial. But often, the age-related differences were not in the direction we hypothesized.

Regarding physical outcomes, consistent with our hypotheses, the transition group did report fewer physical challenges via comorbidities and non-SCD somatic complaints. But contrary to our hypotheses, we found few differences in hemoglobin or white cell count, other than higher HbS. This is somewhat different than McKerrell et al. [60] who, comparing patients under 30 to patients over 40, found significantly less evidence of organ damage in younger patients: higher hemoglobin and platelet counts, higher creatinine clearances, and lower BUN levels.

Also contrary to our hypotheses, the transition group reported less frequent pain, pain in fewer locations, and utilized opioids less often when in pain than either adult groups. Interestingly, pain intensity did not differ significantly between age groups on either crisis or noncrisis pain days. This agreed with the results of Ahmed et al. [59].

Considering utilization, consistent with our disruption hypotheses, the transition group made fewer ambulatory visits unadjusted for pain days and number of comorbidities. In addition, after adjustment, those in the transition group were more likely to use ED/hospital resources to manage a crisis than the older groups. An additional explanation for this finding involves access to insurance. Minors with SCD are likely to be on Medicaid [31] but lose eligibility when they become adults. In Virginia, this occurs at the age of 18. To qualify for Medicaid as adults, one must meet severe income limits and either be pregnant or be a parent or relative caretaker of dependent children.

When considering psychosocial and behavioral outcomes, despite more unplanned visits, the transition group reported less depression/anxiety, less behavioral coping, and better HRQOL than older patients. This was consistent with our hypotheses. In the case of behavioral coping strategies, the differences were strong and remained after adjustment. We have previously reported that catastrophizing did not differ by age [61].

We also tested our hypotheses comparing younger (but posttransition age) adults to older adults. Contrary to our hypotheses, younger adults did not have less pain or better psychosocial function that older adults. Surprisingly, there appeared to be a (nonsignificant) tendency for younger adults to have more ED visits and hospitalizations than either the transition group or older adults, who looked similar to each other. Further, contrary to our hypotheses, based on other differences in HRQOL, such as gender differences in PiSCES [62] and childhood versus adult HRQOL in other studies [23, 63, 64], we did not find that older adults reported poorer functional status and utilized services more often than younger adults. Older adults had higher comorbidity, but fewer ED and hospital visits, suggesting that older adults group were not faring worse than the younger adults after all.

Our findings can also be compared to those of Sanders et al. [58] who studied age differences in adults but only studied a single younger age group: 18–36, versus patients aged 37 and older. Similar to their findings, we found no differences in pain intensity during crisis or noncrisis days. Unlike Sanders et al., though, we found no significant differences in ED visits and hospitalizations. In fact, numerically it appeared that the youngest people with SCD had similar number of ED visits as the oldest adult group. Results from the CSQ-SCD also differed, as we did not find the same differences (ignoring pain, use of heat, and praying), while finding other differences instead (calm, isolation, drinking, and resting). Subdividing their younger group into a transition group and a young adult group as we did appeared to reveal interesting differences in findings for the transition group, who are still developing both physically, emotionally, and socially, as well as for those who were “old” for a SCD patient. The demographics of their sample were similar in terms of marital status and education, although they had slightly more females (68% versus our 60%) and slightly more patients with SS (81% versus our 71%), generally considered the more severe genotype. It is possible that their higher percentage of SS patients accounted for some differences between results, but the differences in percentage were small.

Ahmed et al. used similar age categories when comparing SF-36 quality of life measures for a sample of people with sickle cell disease in Saudi Arabia [59]. Contrary to our findings, they did not find differences in physical function across any age category, but they did find differences in mental health, with the younger group reporting better mental health. Most quality of life measures differentiated functioning in children (up to age 17) as compared to adults of any age. There appeared to be a trend for improvement with emotional role limitations with age, which is the reverse of our sample where those aged 16–25 had better emotional role functioning [59]. Differences in results could be cultural, as Ahmed et al. enrolled people from Saudi Arabia. Additionally, their sample also had more males (58.8% versus 38% in our sample). We previously found that QOL differed by gender, with males having higher (more favorable) results [47]. Ahmed et al. also found that family support was poorer for older adults, while we found no differences in age groups. Family support could potentially relate to reported mental health.

Blinder et al. [31] found that ED reliance increased until age 22 and then remained high but relatively stable. In contrast, in PiSCES, the ED reliance was very similar for patients in the 16–25 and 26–36 year age groups and then decreased by 50% for patients over age 36. This did not change when controlling for pain and comorbidity. A similar pattern to PiSCES was found in Hemker et al. [29], that is, the number of ED visits and the ED ratio were similar in transition-aged and young adults but then decreased in adults 31–45 years old. The ED reliance for health care is supposed to be able to distinguish between increased need versus lack of adequate access to primary care. The decrease in ED visits for older adults could also be due to choice. These older adults were more likely to treat their crises at home, which is consistent with lower EDR for health care. One possible reason for this would be the avoidance of the ED due to stigma developed with repeated ED visits over the years which might negatively impact their desire to utilize the ED [65]. It should be noted that both Blinder et al. [31] and Hemker et al. [29] followed SCD patients with Medicaid in selected states, which means that their results apply only to patients with some insurance coverage.

We did not find differences between age groups on specialty care, with about 50% in specialty care across all age groups. Thus even our youngest group, while transitioning to adult care, still received specialty care at the same rate as the others. This may have mitigated some of the findings that others found for utilization, where specialty care may not have been as common.

Ours was not a longitudinal study. As such, age differences we found could be due to cohort effects, rather than specific age effects. Survival of only the “fittest” SCD patients to an older age could explain some of the characteristics of the older patient group. Also, pain and utilization summary estimates were based on as few as 30 days and as many as 188 days of diaries. Since pain may be episodic in SCD, some important pain episodes could have been missed by our diary methods. If this differed by age group it would bias comparisons. We have no reason to believe, though, that this missing data would differ by age. In fact, the distribution of number of diary days was similar in the 3 age groups (transition: 139 days; younger adults: 125 days; older adults: 138 days). Further, our study did not include children, so we could make no comparisons of children versus adults, which would have been useful to further explore our adolescent disruption hypothesis

## 5. Conclusion

Many of our hypothesized age-associated differences in biological, psychosocial, quality of life, and utilization outcomes in adults with SCD were confirmed. In general, older age patients have worse biological, psychological, and HRQOL outcomes than transition-age patients. In some instances, the oldest patients are not the worst with regard to these outcomes. In contrast, while transition-aged SCD patients do not have worse biology than older patients, they do have more utilization for the amount of pain and biological abnormalities seen, compared to older adults. This sheds some light and adds some validity to current interventions focused on transition-aged patients. But further study is required to determine whether analogous, age-appropriate health care or other interventions could somehow improve the quantity and quality of life for older, posttransition patients with SCD.

## Supplementary Material

Supplementary tables provide additional information on comorbidities and health related quality of life. Specifically, Supplemental Table 1 has detailed SCD comorbidity information (frequencies and percentages) by age group, along with a comparison across age groups. Supplemental Table 2 has means and standard deviations of the eight SF-36 subscales, along with a comparison across age groups.

## Figures and Tables

**Table 1:** Demographic, biological variables, depression, anxiety, and alcohol abuse^1^.

Variable	Transition group (ages 16–25) *N* = 57	Younger adults (ages 26–36) *N* = 71	Older adults (ages 37–64) *N* = 104	*p* value
Gender				0.936
Male	23 (40.3)	27 (38.0)	39 (37.5)	
Female	34 (59.7)	44 (62.0)	65 (61.0)	
Education				0.008^ab^
<High school	13 (22.8)	5 (7.0)	10 ( 9.6)	
High school	26 (45.6)	26 (36.6)	36 (34.6)	
>High school	18 (31.6)	40 (56.3)	58 (55.8)	
Marital status				<0.001^abc^
Married	1 (1.7)	12 (16.9)	42 (40.4)	
Unmarried	56 (98.3)	59 (83.1)	62 (59.6)	
Income				0.972
≤10,000	22 (41.5)	27 (38.0)	39 (37.9)	
10,000–20,000	11 (20.7)	17 (23.9)	24 (23.3)	
20,001–30,000	6 (11.3)	12 (16.9)	16 (15.5)	
>30,000	14 (26.4)	15 (21.1)	24 (23.3)	
Genotype				0.793
S*β*^0^ Thal	1 (1.7)	1 (1.4)	3 ( 2.9)	
S*β*^+^ Thal	1 (1.7)	2 (2.9)	3 ( 2.9)	
SC	10 (17.5)	17 (24.3)	29 (27.9)	
SS	45 (78.9)	50 (71.4)	69 (66.3)	
Seen at SCD specialty center				0.788
Y	27 (49.1)	35 (51.5)	48 (46.1)	
N	39 (50.9)	36 (48.5)	56 (53.9)	
Number of comorbidities	1.9 (0.2)	2.1 (0.2)	3.0 (0.2)	<0.001^bc^
Depression/anxiety				0.014^ab^
Y	8 (14.0)	26 (36.6)	33 (31.7)	
N	49 (86.0)	45 (63.4)	71 (68.3)	
Alcohol abuse				0.419
Y	14 (24.6)	22 (31.0)	36 (34.6)	
N	43 (75.4)	49 (69)	68 (65.4)	
Lab values^2^				
% F	5.1 (1.1)	2.9 (1.0)	3.9 (0.8)	0.319
HbA	7.7 (2.9)	13.2 (2.6)	13.3 (2.2)	0.262
HbC	8.8 (2.9)	10.9 (2.6)	13.0 (2.1)	0.485
HbS	78.3 (2.9)	71.6 (2.1)	65.8 (2.2)	0.003^b^
HCT	26.3 (0.8)	28.2 (0.7)	27.1 (0.6)	0.238
WBC	11.4 (0.7)	11/0 (0.6)	10.7 (0.5)	0.726

^1^Frequency (%) or mean (SD).

^2^Reduced sample size for labs (*n* = 193, 201, 200, 212, 203, and 169 for % F, HbA, HbC, HbS, HbS, HCT, and WBC).

Multiple comparisons (Bonferroni, Tukey): significant differences in unadjusted analyses, transition versus younger adults ^a^; transition versus older adults ^b^; younger versus older adults ^c^.

**Table 2:** Pain-related variables (unadjusted means and standard errors).

Variable	Transition group (ages 16–25) *N* = 57	Younger adults (ages 26–36) *N* = 71	Older adults (ages 37–64) *N* = 104	*p* value for overall ANOVA
% Pain days	32.7 (4.8)	62.4 (4.3)	64.7 (3.6)	<0.001^ab^
% Crisis days	8.4 (3.0)	18.3 (2.7)	17.0 (2.3)	0.033^a^
Mean pain intensity^*∗*^				
Pain days	4.2 (0.2)	4.3 (0.2)	4.4 (0.2)	0.687
Crisis days	5.6 (0.3)	5.4 (0.2)	5.5 (0.2)	0.867
Noncrisis pain days	3.8 (0.2)	3.9 (0.2)	4.1 (0.2)	0.451
Mean # body parts that hurt	2.4 (0.3)	3.2 (0.2)	3.6 (0.2)	0.001^b^

Multiple comparisons (Bonferroni, Tukey): significant differences in unadjusted analyses, transition versus younger adults ^a^; transition versus older adults ^b^; younger versus older adults ^c^.

^*∗*^Mean pain intensity, measured on days when pain was reported, varies from 1 to 9, with higher values implying more pain.

**Table 3:** Health care utilization (unadjusted means and standard errors).

Variables	Transition group (ages 16–25) *N* = 57	Younger adults (ages 26–36) *N* = 71	Older adults (ages 37–64) *N* = 104	*p* value for overall ANOVA
% Home pain days on opioids^*∗*^	50.1 (5.3)	62.1 (4.5)	70.9 (3.8)	0.007^b^
% Days with ED visits	1.1 (0.4)	2.2 (0.4)	1.1 (0.3)	0.079
% Days in hospital	1.9 (0.8)	3.0 (0.7)	1.8 (0.6)	0.365
% Days with outpatient visit	1.5 (0.6)	2.7 (0.6)	3.6 (0.5)	0.029^b^
% Days with any utilization	3.7 (1.2)	6.9 (1.1)	5.9 (0.9)	0.148
ED reliance-health care^*∗∗*†^	36.7 (4.8)	39.6 (4.2)	19.3 (3.4)	<0.001^bc^
ED reliance-crisis^†^	19.8 (4.0)	14.2 (3.3)	10.6 (2.8)	0.171

Multiple comparisons (Tukey): significant differences in unadjusted analyses, transition versus younger adults ^a^; transition versus older adults ^b^; younger versus older adults ^c^.

^*∗*^6 pts in the Transition Group, 1 in the younger adults, and 6 in the older adult groups did not have any home pain days, so are missing home days on opioids

^*∗∗*^14 pts in the Transition Group, 18 in the younger adult, and 23 in the older adult group did not have either outpatient or ED visits reported on their diary, so ED reliance could not be computed.

† represents percentage of ambulatory visits cared for in ED rather than outpatient or percentage of crises cared for at the hospital rather than at home. Higher values imply more reliance on the ED/hospital.

**Table 4:** Psychosocial variables, HRQOL^*∗*^ (unadjusted means and standard errors).

	Transition group (ages 16–25) *N* = 57	Younger adults (ages 26–36) *N* = 71	Older adults (ages 37–64) *N* = 104	*p* value for overall ANOVA^*∗∗*^
Somatic symptom score	5.4 (0.5)	7.3 (0.4)	7.9 (0.4)	<0.002^ab^
Coping^†^ (CSQ-SCD)				
Active	2.6 (0.1)	2.9 (0.1)	3.0 (0.1)	0.078
Affective/emotional focused	2.1 (0.2)	2.6 (0.1)	2.5 (0.1)	0.058
Passive/behavioral adherence	3.6 (0.1)	4.2 (0.1)	4.0 (0.1)	0.008^a^
HRQOL^††^				
PCS	40.8 (1.3)	34.0 (1.1)	33.0 (0.9)	<0.001^ab^
MCS	49.7 (1.5)	45.5 (1.5)	47.1 (1.1)	0.102
Social support	5.5 (0.2)	5.5 (0.2)	5.9 (0.1)	0.112
Stress	18.3 (1.34)	21.1 (1.2)	19.4 (1.0)	0.263

^*∗*^Higher scores are better, except for the stress measure.

^*∗∗*^Multiple comparisons (Tukey): significant differences in unadjusted analyses, transition versus younger adults ^a^; transition versus older adults ^b^; younger versus older adults ^c^.

^†^Coping is measured using the Coping Strategies Questionnaire-SCD (CSQ-SCD).

^††^HRQOL = Health Related Quality of Life, Measured using MOS SF-36: PCS = physical summary score. MCS = mental summary score.
