# Supplementary material for: Comorbidity, Pain, Utilization, and Psychosocial Outcomes in Older versus Younger Sickle Cell Adults: The PiSCES Project
Source: Biomed Res Int. 2017 Mar 28;2017:4070547. doi: 10.1155/2017/4070547 (PMC5387810; doi:10.1155/2017/4070547)
Supplement: Supplementary file 1 — Supplementary tables provide additional information on comorbidities and health related quality of life. Specifically, Supplemental Table 1 has detailed SCD comorbidity information (frequencies and percentages) by age group, along with a comparison across age groups. Supplemental Table 2 has means and standard deviations of the eight SF-36 subscales, along with a comparison across age groups. [file 4070547.f1.docx]

Supplemental Material

Supplemental table 1. Comparison across age of specific comorbidities

| Comorbidities | *Ages 16-25*  *N=57* | *Ages 26-36*  *N=71* | *Ages 37-64*  *N=104* | *p-value* |
| --- | --- | --- | --- | --- |
| Gout | 0 (0) | 6 (8.4) | 17 (16.5) | 0.0033^b^ |
| AVN | 4 (7.0) | 15 (21.1) | 29 (28.2) | 0.0068^b^ |
| IschemicUlcers | 1 (1.7) | 4 (5.6) | 21 (20.4) | 0.0003^bc^ |
| Kidney | 1 (1.7) | 3 (4.2) | 6 ( 5.8) | 0.4793 |
| Stroke_TIA | 8 (14.0) | 8 (11.3) | 14 (13.5) | 0.8771 |
| Priapism | 4 (17.1) | 5 (11.3) | 6 (15.4) | 0.9426 |
| Gallstones | 27 (47.4) | 33 (46.5) | 61 (59.2) | 0.1739 |
| Osteomyelitis | 3 (5.3) | 0 (0) | 8 ( 7.8) | 0.0598 |
| UTI | 22 (38.6) | 32 (45.1) | 46 (44.2) | 0.7269 |
| Meningitis | 1 (1.7) | 1 (1.4) | 3 (2.9) | 0.7691 |
| Sepsis | 1 (1.7) | 1 (2.8) | 7 (6.9) | 0.2311 |
| Abscess | 2 (3.5) | 3 (4.2) | 6 ( 5.9) | 0.7701 |
| Sarcoidosis | 0 | 0 | 2 (1.9) | 0.2890 |
| Lupus | 0 | 0 | 2( 1.9) | 0.2855 |
| CHF | 2 (3.5) | 3 (4.2) | 13 (12.7) | 0.0457 |
| Asthma | 9 (6.1) | 10 (14.1) | 10 (9.6) | 0.4492 |
| Hypertension | 2 (3.5) | 2 (2.8) | 17 (16.4) | 0.0022^bc^ |
| Rheumatism | 2 (3.5) | 4 (5.6) | 20 (19.2) | 0.0021^bc^ |
| Lung Problems | 18 (31.6) | 20 (28.2) | 2 (21.6) | 0.3445 |

*Multiple comparisons (Bonferonni): Significant differences in unadjusted analyses, transitionvs younger adults (a); transition vs older adults (b); younger vs older adults (c).

Supplemental Table 2: SF-36 subscales (unadjusted means and standard errors)

| Scale | Ages 16-25  N=57 | Ages 26-36  N=71 | Ages 37-66  N=104 | p-value |
| --- | --- | --- | --- | --- |
| General Health | 48.7 (2.8) | 35.4 (2.5) | 38.1 (2.1) | 0.0014^ab^ |
| Vitality | 50.6 (3.0) | 35.5 (2.6) | 41.6 (2.2) | 0.0009^ab^ |
| Bodily Pain | 58.7 (3.5) | 43.2 (3.1) | 45.8 (2.6) | 0.0024^ab^ |
| Mental Health | 75.7 (2.7) | 69.5 (2.4) | 72.6 (2.0) | 0.2262 |
| Physical Function | 75.1 (3.1) | 61.7 (2.8) | 54.3 (2.3) | <0.0001^ab^ |
| Role Physical | 59.2 (5.1) | 36.3 (4.5) | 32.0 (3.7) | <0.0001^ab^ |
| Role Emotional | 77.6 (5.6) | 58.1 (4.9) | 52.5 (4.1) | 0.0015^ab^ |
| Social Function | 71.5 (3.4) | 61.3 (3.1) | 60.7 (2.6) | 0.0298^b^ |

multiple comparisons (Tukey): ^a^significant differences between ages 16-24 and 25-36;  ^b^significant differences between ages 16-24 and 37-64; ^c^significant differences between ages 25-36 and 37-64

Age group differences no longer significant after controlling for pain days and number of comorbidities: General health (44.0, 36.3, 40.1, p=0.1213), Bodily Pain (51.1, 44.6, 49.0, p=0.2933) ; Role Emotional (71.4, 59.6, 54.8, p=0.0818) and Social Function(66.4, 62.5, 62.7, p=0.6552).
